# Nitrogen-Tungsten Oxide Nanostructures on Nickel Foam as High Efficient Electrocatalysts for Benzyl Alcohol Oxidation

**DOI:** 10.3390/molecules29163734

**Published:** 2024-08-07

**Authors:** Yizhen Zhu, Xiangyu Chen, Yuanyao Zhang, Zhifei Zhu, Handan Chen, Kejie Chai, Weiming Xu

**Affiliations:** 1 College of Material, Chemistry and Chemical Engineering, Hangzhou Normal University, Hangzhou 311121, China; 2 Kharkiv Institute, Hangzhou Normal University, Hangzhou 311121, China

**Keywords:** WO-N/NF electrode, benzyl alcohol, nitrogen-doped bimetallic oxide electrocatalyst, nickel foam, electrooxidation

## Abstract

Electrocatalytic alcohol oxidation (EAO) is an attractive alternative to the sluggish oxygen evolution reaction in electrochemical hydrogen evolution cells. However, the development of high-performance bifunctional electrocatalysts is a major challenge. Herein, we developed a nitrogen-doped bimetallic oxide electrocatalyst (WO-N/NF) by a one-step hydrothermal method for the selective electrooxidation of benzyl alcohol to benzoic acid in alkaline electrolytes. The WO-N/NF electrode features block-shaped particles on a rough, inhomogeneous surface with cracks and lumpy nodules, increasing active sites and enhancing electrolyte diffusion. The electrode demonstrates exceptional activity, stability, and selectivity, achieving efficient benzoic acid production while reducing the electrolysis voltage. A low onset potential of 1.38 V (vs. RHE) is achieved to reach a current density of 100 mA cm^−2^ in 1.0 M KOH electrolyte with only 0.2 mmol of metal precursors, which is 396 mV lower than that of water oxidation. The analysis reveals a yield, conversion, and selectivity of 98.41%, 99.66%, and 99.74%, respectively, with a Faradaic efficiency of 98.77%. This work provides insight into the rational design of a highly active and selective catalyst for electrocatalytic alcohol oxidation.

## 1. Introduction

In recent times, the depletion of fossil fuels and the pressing concern for environmental pollution have led to a surge in interest in generating renewable and clean energy sources [1,2,3,4]. Hydrogen energy is considered a promising energy source to substitute fossil fuels and provide a viable solution to the energy crisis and environmental issues [5,6]. Water electrolysis has a promising future for renewable energy in the hydrogen production industry, owing to its eco-friendly and sustainable characteristics [7,8]. However, there are still some limitations to large-scale hydrogen production via water electrolysis [9,10]. Firstly, the overpotentials required for the anodic oxygen evolution reaction (OER) and cathodic hydrogen evolution reaction (HER) throughout the water electrolysis procedure are excessive, consequently resulting in higher energy consumption [11,12]. In addition, the economic value of the anode by-product oxygen is low in the traditional approach of water electrolysis [13].

In light of the challenges associated with conventional water electrolysis, utilizing electrocatalytic organic oxidation reactions emerges as a more viable alternative [12,14]. Specifically, substituting the OER with the electrocatalytic oxidation of benzyl alcohol not only reduces the required potential for water electrolysis but also yields benzoic acid, a high-value chemical pivotal in both industrial production and fundamental research. This green approach, integrating electrocatalytic alcohol oxidation with hydrogen production, presents distinct advantages, including a lower potential, prevention of gas mixing, and the generation of value-added products. These merits position it as a promising research focus in the field [15,16]. Electrocatalytic oxidation technology leverages variations in electrode potential to drive electron transfer, expediting the synthesis of organic compounds. This approach avoids the use of additional toxic reagents, thereby reducing environmental pollution. It is also in line with the principles of sustainable development within modern industry and environmental conservation. Notably, the prevalent use of noble metals (e.g., Pt, Ir, Rh, Ru, etc.) or their oxides as electrode catalysts in electrochemistry faces limitations in commercial applications due to their prohibitively high costs [16].

As a three-dimensional (3D) porous substrate, nickel foam exhibits remarkable electrical conductivity, facilitating electron diffusion. Additionally, its high specific surface area promotes the even distribution of catalysts, and its porous structure enables the swift diffusion of gases that are produced. Xu and coworkers employed in situ construction of NiCo_2_O_4_ nanosheet catalysts on nickel foam for electrocatalytic benzyl alcohol oxidation reactions, which required a mere 1.46 V (vs. RHE) to achieve a current density of 100 mA·cm^−2^. After 2 h of reaction under optimized experimental conditions, the NiCo_2_O_4_/NF catalyst achieved over 95% conversion of benzyl alcohol and demonstrated high selectivity to benzoic acid, as well as high Faradaic efficiency and excellent stability [17]. Moreover, transition metal tungstates (TMTs) containing abundant element tungsten (W) are regarded as one of the most promising electrode materials for electrochemical energy storage compared to noble metal electrode materials [18]. Compared to traditional metals, tungsten (W) offers superior stability and extended service life in electrochemical applications, enhancing its appeal as an electrocatalyst. Tungsten-based materials also retain their performance under harsh electrochemical conditions, unlike other metals, which may degrade more quickly in strong oxidative environments [19,20]. TMTs manifest high durability, cost-effectiveness, and environmental compatibility [21]. However, TMTs face challenges in capacity and stability due to low electrical conductivity and nanoparticle aggregation [22]. Therefore, a common approach to address these issues involves synthesizing dispersed nanocomposites with enhanced electrical conductivity and a strategy supported by studies showing substantial improvement in electrocatalyst efficiency through nitrogen (N) doping [23,24,25]. This strategy not only enhances the material’s electrical conductivity but also fortifies the stability of nickel foam [26,27]. For instance, Wang and co-workers loaded a novel copper-nickel nitride (Cu_1_Ni_2_-N) which was enriched with a Cu_4_N/Ni_3_N interface on carbon fibers [28]. As a result, the electrode demonstrated a hydrogen precipitation overpotential of 71.4 mV at a current density of 10 mA·cm^−2^ and exhibited outstanding stability over 75 h. However, current research primarily focuses on improving the OER reaction activity, with limited exploration of TMT materials for electrocatalytic alcohol oxidation and hydrogen production.

In this work, a one-step hydrothermal method was employed to synthesize WO-N/NF using nickel foam and ammonium metatungstate. The resulting product was effectively used in the catalytic oxidation reaction of benzyl alcohol. The impact of varied solution concentrations and hydrothermal reaction times on the electrodes was explored. The microscopic morphology, crystal structure, elemental composition and content, and elemental valence states of the synthesized electrodes’ surface were comprehensively investigated with scanning electron microscopy, X-ray diffraction, energy dispersal spectroscopy, and X-ray photoelectron spectroscopy. The hydrogen precipitation performance of the electrode in alkaline electrolytes was characterized with the aid of an electrochemical workstation. The optimal hydrothermal reaction time and reaction solution concentration were considered during the analysis.

## 2. Results and Discussion

### 2.1. Characterization of WO-N/NF

**Figure 1:**
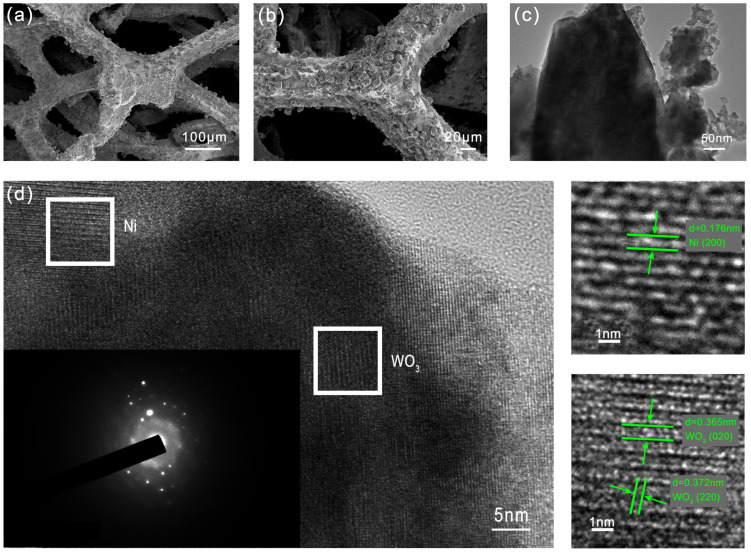
Surface morphology characterization of WO-N/NF electrode: (**a**,**b**) SEM; (**c**) TEM, and (**d**) HRTEM image and SAED pattern (inset in (**d**)).

**Figure 2:**
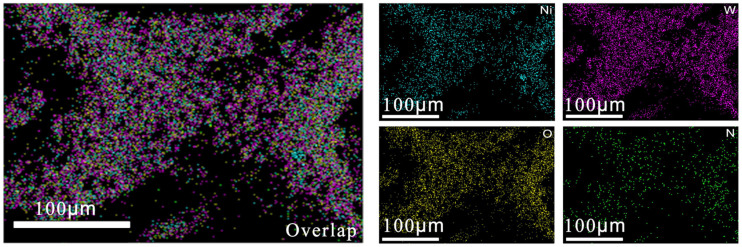
EDS mapping images of the WO-N/NF nanocomposite.

WO-N nickel foam electrodes were synthesized via a one-step hydrothermal approach, utilizing nickel foam as the substrate and ammonium metatungstate as the nitrogen and tungsten sources. The hydrothermal reaction induced a discernible change in the surface color of the Ni foam, transitioning from silver to yellowish green (Figure S1), which implies a new substance was produced. As the SEM images showed (Figure 1a, Figure S2), the coating grows directly on the nickel foam substrate, and its surface shows inhomogeneity and roughness accompanied by the presence of cracks. The formation of these cracks may be associated with the presence of residual stresses within the coating [29]. In addition, lumpy nodules appeared on the surface of the electrode, increasing the specific surface area of the electrode. This structure helps provide more active sites and increases the contact area between the electrolyte and the electrode surface, facilitating electrolyte diffusion. As can be seen from the higher magnification images (Figure 1b), the surface of the WO-N/NF electrode is characterized by block-shaped particles. It is observed that these particles are grown on the coating with a distributed pattern, which may suggest a staggered arrangement of nanosheets [30]. This direct-grown block coating avoids the use of adhesives, thereby reducing the distance of diffusive electron transport and lowering the electron transfer resistance, thereby facilitating the oxidation reaction. TEM imaging of the powders obtained from the Ni foam substrate further validated the morphology of the sheet arrays (Figure 1c). The high-resolution TEM image (Figure 1d) reveals the presence of three lattice fringes with a spacing of 0.365 nm, 0.372 nm, and 0.176 nm, which can be attributed to the (220) and (020) lattice planes of WO_3_ and the (200) lattice plane of Ni, respectively. The corresponding SAED pattern (Figure 1d) shows well-defined spots, which can be readily indexed to monoclinic WO_3_ [19]. In addition, the elemental mapping image (Figure 2) reveals a uniform distribution of Ni, W, O, and N on the electrode surface. To determine the elemental composition and content of the WO-N/NF electrode’s surface, EDS analysis was conducted. The EDS spectrum in Figure S3 and Table S1 provides information on the composition of the WO-N/NF electrode, allowing for the determination of the presence and content of each element.

**Figure 3:**
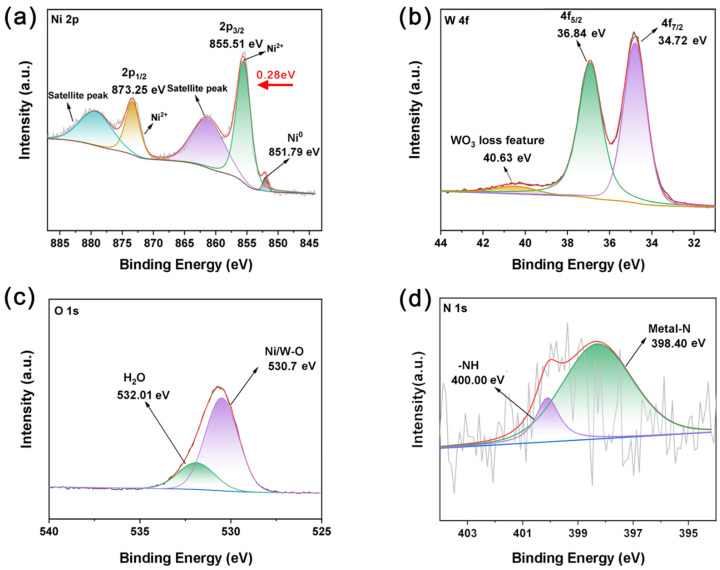
XPS spectrums of Ni 2p (**a**), W 4f (**b**), O 1s (**c**), and N 1s (**d**) for the obtained WO-N/NF electrode.

XPS was utilized to analyze the surface composition and elemental chemistries of the WO-N/NF electrode. Figure S4 confirmed the presence of Ni, W, O, and N, consistent with the EDS results. The high-resolution spectrum of Ni 2p (Figure 3a) shows double peaks at binding energies of 855.51 eV and 873.25 eV, indicating the presence of nickel in the Ni^2+^ state. Notably, two satellite peaks at 861.39 eV and 879.23 eV are observed [31,32,33]. A minor peak at 851.79 eV corresponds to the 2p_3/2_ orbital of Ni^0^ from the nickel foam substrate, indicating that certain fragments on the nickel foam surface have oxidized to Ni^2+^. Higher valence Ni effectively reduces the reaction potential required for its oxidation [34]. As depicted in Figure S5, there are double peaks with binding energies of 855.23 eV and 872.97 eV, indicating the presence of nickel in the Ni^2+^ state. Compared with hydrothermal NF, it is noteworthy that this double peak also appears in the WO-N/NF sample after W doping, with the corresponding binding energy shifted by 0.28 eV to the higher energy region. This result indicates the successful doping of W elements and shows that the doping affects the electronic structure of the catalyst material. It has been reported that metal nitrides are easily oxidized during the oxygen evolution reaction to produce the highly reactive species NiOOH [35,36]. Therefore, the doping of W causes the Ni in the WO-N/NF samples to lose electrons, creating an electron-deficient state that is more likely to produce more reactive species (NiOOH), thus promoting the activity of the hydrogen evolution reaction. The high-resolution energy spectrum of W 4f (Figure 3b) exhibited two primary peaks at binding energies of 36.84 eV and 34.72 eV, attributed to W4f_5/2_ and W4f_7/2_, respectively, indicating the presence of W^6+^. In addition, the small peak on the left with a binding energy of 40.63 eV represents W 5p_3/2_. According to the literature, W^6+^ is predominantly present in catalysts such as WO_3_ or WO_2_^4−^ [37,38,39,40]. In the O 1s high-resolution energy spectrum (Figure 3c), the peak at 532.01 eV corresponds to water molecules adsorbed on the electrode surface, while the peak at 530.7 eV is attributed to the Ni/W-O bond [29]. The N 1s spectrum (Figure 3d) exhibits two peaks at 398.40 eV and 400.00 eV, corresponding to the Ni/W-N and -NH bonds, respectively [41,42,43]. The above experimental results show that the one-step hydrothermal method successfully prepared nickel foam electrodes doped with tungsten and nitrogen elements.

**Figure 4:**
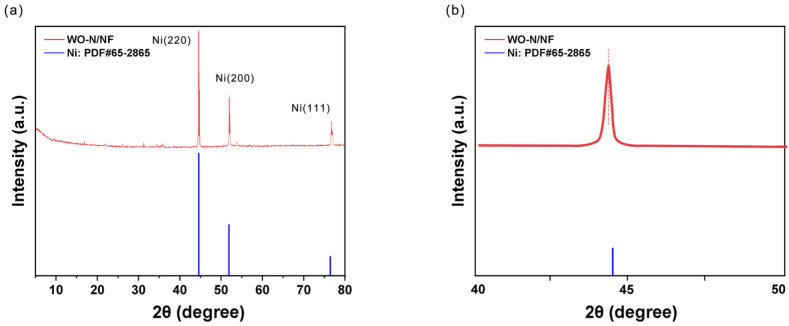
Structure test diagram of electrodes: (**a**) XRD patterns of WO-N/NF; (**b**) localized magnified XRD patterns at 40° to 50°.

XRD analysis was conducted to probe the phase state of the sample. As shown in Figure 4a, the electrode exhibits three major diffraction peaks at 44.70°, 52.02°, and 76.64°, corresponding to the (111), (200), and (220) crystal planes of metallic Ni (JCPDS No. 65-2865). The thin thickness of the hydrothermal accumulation layer may contribute to the full display of the nickel foam peaks within the matrix. In Figure 4b, the local magnification of the electrode from 40° to 50° reveals a leftward shift of the WO-N/NF electrode relative to the standard card on the Ni (111) surface. According to the Bragg equation, this shift suggests the solid dissolution of W and N atoms in the Ni lattice, precluding the appearance of W- and N-related diffraction peaks [44,45].

### 2.2. Electrocatalytic Performance

To better investigate the catalytic oxidation activity of the WO-N/NF electrode, a standard three-electrode system with a platinum (Pt) electrode as the counter electrode and Ag/AgCl as the reference electrode was used in 1 M KOH alkaline medium using an electrochemical workstation at a scan rate of 40 mV s^−1^.

**Figure 5:**
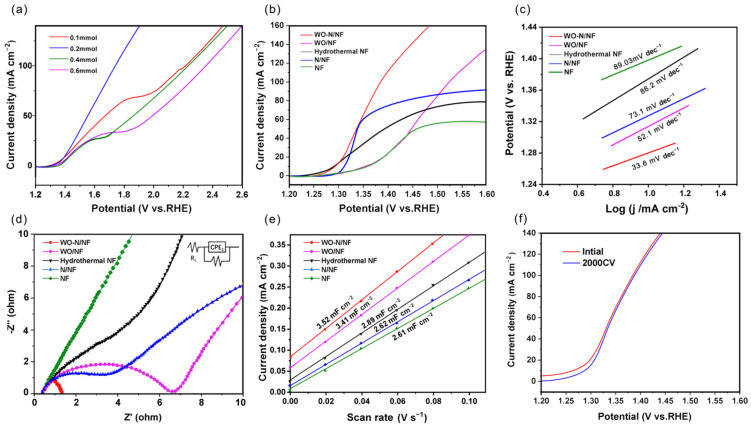
(**a**) LSV plots for WO-N/NF with different concentrations of precursors. (**b**) LSV curves of WO-N/NF, WO/NF, N/NF, hydrothermal NF and NF at the anode and (**c**) Tafel plots derived from (**b**); (**d**) Nyquist diagrams (the inset is an equivalent circuit model); (**e**) double layer capacitance diagram; (**f**) anodic polarization curves before and after 2000 CV.

Initially, to determine the appropriate amount of doping in WO-N/NF, we conducted Linear Sweep Voltammetry (LSV) to assess its electrocatalytic performance across different concentrations. Figure 5a illustrates the LSV curves for nickel foam electrodes with varied W-doped concentrations in 40 mL of 1.0 M KOH with 0.1 M benzyl alcohol electrolyte. Notably, the 0.2-WO-N/NF displays the most pronounced slope, indicative of accelerated current density growth, minimal alterations in overpotential, and superior electrocatalytic performance. Consequently, we designate 0.2-WO-N/NF as the optimal doping level. After this, a comprehensive analysis of the electrochemical properties of the WO-N/NF electrodes was conducted, encompassing cyclic voltammetry, linear scanning voltammetry, and electrochemical impedance spectroscopy.

For comparison, the catalytic activities of WO-N/NF, WO/NF, N/NF, hydrothermal NF, and bare NF were also measured under the same conditions. As depicted in the polarization curves (Figure 5b), the slope of the WO-N/NF electrode exceeds that of the other electrodes in an alkaline electrolyte containing benzyl alcohol, indicating superior catalytic oxidation performance [46,47]. Furthermore, the LSV curve of the WO-N/NF electrode was measured with and without 0.1 M benzyl alcohol (Figure S6). The electrode requires 1.52 V (vs. RHE) to achieve a current density of 20 mA cm^−2^ without benzyl alcohol, outperforming the benchmark electrolyzer consisting of a Pt-C/NF cathode and a RuO_2_/NF anode, which requires 1.60 V to reach the same current density [48]. The Tafel slope, a crucial parameter for evaluating electrode catalytic performance, was determined using the Tafel equation η=blogj+a, where a and b denote the intercept and Tafel slope, respectively. Figure 5c depicts the Tafel plots of the corresponding polarization curves. In this work, the slope for WO-N/NF is 33.6 mV dec^−1^, which is significantly lower than that of WO/NF (52.1 mV dec^−1^), N/NF (73.1 mV dec^−1^), hydrothermal NF (86.2 mV dec^−1^), and NF (144 mV dec^−1^), indicating a preferable HER kinetics on WO-N/NF. Generally, a smaller Tafel slope indicates a greater ability of the electrode material to transfer charge and a faster reaction rate of the electrode [25]. Thus, the WO-N/NF electrode exhibits a faster oxidation reaction rate.

Electrochemical Impedance Spectroscopy (EIS) tests were conducted to investigate the internal resistance and charge transfer at the surface of samples. Figure 5d illustrates the EIS profiles of the WO-N/NF and other electrodes under bias. All electrodes display a semicircular shape in their impedance graphs, with the WO-N/NF electrode exhibiting a markedly smaller radius than the others. Additionally, the equivalent circuit model used for the EIS measurements is shown, consisting of three elements: Rs, Rct, and CPE. These represent the solution resistance of the alkaline electrolyte, the charge transfer resistance at the electrode–electrolyte interface, and the constant phase element of the bilayer at the same interface, respectively. Zview software (Version 3.1) was used to fit the EIS data using this equivalent circuit. The fitting results, as detailed in Table 1, indicate that the charge transfer resistance of WO-N/NF is 1.36 Ω, which is lower than that of WO/NF (6.84 Ω), N/NF (8.3 Ω), hydrothermal NF (97.21 Ω), and NF (590.02 Ω). This suggests that ion diffusion in the electrolyte is faster and that the HER kinetic process is more rapid on WO-N/NF. Generally, a smaller charge transfer resistance translates to lower electron resistance during the transfer process, facilitating faster electron transfer and oxidation rates, ultimately leading to improved electrode oxidation performance [49]. The WO-N/NF electrode exhibits a faster oxidation reaction rate and better oxidation catalytic performance due to its significantly smaller charge transfer resistance compared to the other electrodes [50]. According to the Engel–Brewer bonding theory, when the d orbitals of the transition metal element W have an available electron density in excess of the number of d orbitals, alloying with a transition metal such as Ni occurs in the 3d^8^4s^2^ configuration [51,52]. The synergistic interaction between these species contributes to enhancing the performance of the electrode. The incorporation of the W element improved the charge transfer resistance of the Ni electrode and enhanced its oxidation performance. This improvement can be attributed to the electron transfer caused by the introduction of W, which improves the electronic structure of the electrode and enhances its catalytic performance.

The electrochemical active surface area (ECSA) reflects the number of active sites on the electrode’s surface [53]. A greater ECSA implies an increased exposure of active sites, correlating with the heightened catalytic activity of the electrode [53,54]. In Figure 5e, WO-N/NF shows a larger Cdl (3.52 mF cm^−2^) compared to WO/NF (3.41 mF cm^−2^), N/NF (2.62 mF cm^−2^), hydrothermal NF (2.89 mF cm^−2^), and pure NF (2.61 mF cm^−2^). This implies that WO-N/NF contains the most active sites, thereby promoting the HER performance. The ECSA of the electrode can be calculated using the double-layer capacitance, and the Equation (1) for ECSA is as follows:

(1) $$ECSA=\frac{C_{dl}}{C_{s}}$$

The stability and durability of the electrode material are important parameters for evaluating the oxidation performance of the catalyst. In industrial applications, the electrode material must maintain the stability of the morphology and composition of the electrode surface, as well as long-term durability over a prolonged period of operation [54]. Cyclic voltammetry (CV) scanning, a commonly employed technique, accelerates the degradation of electrode materials. Material stability is determined by comparing anodic polarization curves before and after multiple cycles [55]. Figure 5f displays the anodic polarization curves of the WO-N/NF electrode before and after 2000 CV cycles in a 1 M KOH solution at room temperature. The polarization curves after 2000 CV cycles almost coincide with the original polarization curves, indicating that the WO-N/NF electrode exhibits good oxidation performance. Further tests involved chronoamperometry, conducted five times consecutively for four hours at a potential of 0.77 V (vs. Ag/AgCl) (Figure S7). This potential was chosen as it ensures no significant anodic oxygen evolution reaction. The figure demonstrates that the electrolysis curve of the WO-N/NF electrode maintains stability in 1M KOH electrolyte, indicating good stability. The gradual decrease in electrode current density with electrolysis time, stabilizing after five repeated uses, indicates sustained performance over time.

**Figure 6:**
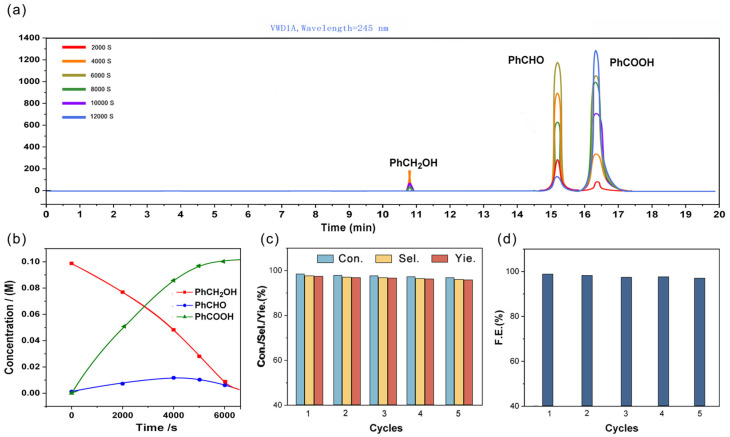
(**a**) HPLC traces of catalysis by WO-N/NF at an actual potential of 0.77 V (vs. Ag/AgCl) in 40 mL of 1.0 M KOH with 0.1 M benzyl alcohol; (**b**) the concentration of benzyl alcohol and its oxidated products during the electrolysis; (**c**) the conversion of benzyl alcohol, and the selectivity and yield of benzoic acid; (**d**) the corresponding faradaic efficiencies for seven electrolysis cycles.

In order to define the products in the process of electrocatalytic oxidation of benzyl alcohol, electrochemical oxidation tests were systematically conducted at a constant potential. Chronoamperometry experiments were repetitively performed over five consecutive sessions, each lasting four hours, at a stable potential of 1.87 V, ensuring negligible occurrence of the OER. The concentrations of benzyl alcohol and its oxidation by-products during the electrolysis process were meticulously tracked using HPLC. Figure 6a shows the high-performance liquid chromatogram of benzyl alcohol during its electrocatalytic oxidation. There are three distinct peaks at different positions corresponding to different electrocatalytic products, which can correspond to benzyl alcohol (peak 1), benzaldehyde (peak 2), and benzoic acid (peak 3), in that order. Meanwhile, a significant amount of hydrogen was discharged from the cathodic electrolytic cell during the electrocatalytic oxidation of benzyl alcohol, verifying that the electrocatalytic oxidation of benzyl alcohol in the anodic electrolytic cell was enhanced. As depicted in Figure 6b, the concentration of benzyl alcohol gradually diminishes throughout the electrochemical oxidation reaction. By the 6000s mark, benzyl alcohol oxidation attains near completion, leading to the quantitative characterization of the acidified anode product. The analysis reveals an impressive yield, conversion, and selectivity of 98.41%, 99.66%, and 99.74%, respectively, coupled with a commendable Faradaic efficiency of 98.77%.

The reusability of the WO-NF catalyst for benzyl alcohol oxidation was investigated. As shown in Figure 6c, after five cycles, the yield, conversion, and selectivity persist at impressive levels of 97.28%, 98.63%, and 98.13%, respectively, with the Faradaic efficiency maintaining a commendable 98.55% (Figure 6d), demonstrating sustained high performance without notable reduction. After five consecutive cycles, no significant changes in the morphology and structure of WO-N/Ni were observed (Figures S8 and S9), indicating its high stability. The Ni 2p and W 4f spectrums (Figure S10) reveal that Ni^2+^/Ni^3+^ and W^6+^ remain the dominant species in WO-N/Ni. Furthermore, the relative contents of metal-O and metal-N do not show significant changes after the reaction. Furthermore, the oxidation reaction follows a first-order reaction, as shown by the fitting lines of −ln(C/C_0_) vs. time in Figure S11 [56,57]. Here, C and t represent the concentration of benzyl alcohol and the oxidation time, respectively. The oxidation rate constants for the WO-N/NF and NF catalysts are 2.11 × 10^−4^ s^−1^ and 1.40 × 10^−4^ s^−1^, respectively. In addition, Table S2 presents a comparative assessment of the catalytic performance of WO-N/NF against previously reported electrodes. These results suggest that the WO-N/NF catalyst exhibits superior catalytic activity in the oxidation of benzyl alcohol compared to the bare NF substrate.

## 3. Experimental Section

### 3.1. Chemicals

Nickel foam was purchased from Kunshan Guangjiayuan New Materials Co., Ltd. (Kunshan, China). Ammonium metatungstate (NH_4_)_6_H_2_W_12_O_40_·XH_2_O was purchased from Shanghai Aladdin Biochemical Technology Co., Ltd. (Shanghai, China). Benzyl alcohol (99%), benzoic acid (99%), and benzaldehyde (99%) were purchased from Energy Chemical (Shanghai, China). Acetone (AR), hydrochloric acid (AR), and acetic acid (AR) were purchased from Sinopharm Chemical Reagent (Shanghai, China). Methanol (HPLC) was purchased from Sinopharm Chemical Reagent and Sigma-Aldrich (St. Louis, MO, USA). All chemical reagents were used without further purification.

### 3.2. Characterization

The crystal structure of the materials was evaluated by X-ray diffraction (XRD, Bruker D8, Bruker, Billarica, MA, USA). The apparent morphologies of the materials and EDS images of the material were investigated using a scanning electron microscope (SEM, Sigma 500, Oberkochen, Germany). Transmission electron microscope (HRTEM, Hillsboro, OR, USA, 200 kV) images were taken using an FEI Tecnai F20 microscope (FEI, Hillsboro, OR, USA). X-ray photoelectron spectroscopy (XPS, Waltham, MA, USA) analysis of the surface electronic states was performed using a Thermo ESCALAB 250Xi (Waltham, WA, USA).

### 3.3. Synthesis of WO-N/NF

The materials were prepared using a one-step hydrothermal method (Figure S12). A 10 × 40 × 1 mm^3^ nickel foam was initially intercepted and then ultrasonically washed in sequence using acetone, 1 M hydrochloric acid, and deionized water for 30 min. Subsequently, the foam was vacuum-dried. A total of 0.2 mmol ammonium metatungstate was dissolved in 40 mL deionized water by stirring at room temperature using a stirring table. The configured solution and nickel foam were moved to a 100 mL hydrothermal reactor and heated in an oven at 160 °C for 8 h then cooled to room temperature; the nickel foam with color change was collected, rinsed with deionized water to remove the impurities on the surface, and dried at 50 °C in a vacuum oven for 12 h to obtain the WO-N/NF. The doping content of W was adjusted by varying the molar amount of ammonium metatungstate. By adding x mmol of ammonium metatungstate, the product obtained was defined as x-WO-N/NF.

### 3.4. Electrochemical Measurement

Electrochemical measurements were carried out using an electrochemical workstation (Autolab PGSTAT128N, Metrohm AG, Herisau, Switzerland). The electrocatalytic oxidation reaction of benzyl alcohol was conducted in an H-type cell double electrolyzer separated by a Nafion-117 proton exchange membrane (Chemours, Wilmington, DE, USA) placed between the two electrolyzers. A three-electrode system was employed in the reaction, utilizing WO-N/NF as the working electrode, a counter electrode composed of a platinum foil measuring 1 cm × 1 cm, and a reference electrode of silver chloride (Ag/AgCl). The measured potentials were converted to reversible hydrogen electrodes using the following equation, Equation (2):

(2) $$E_{\left(vs.RHE\right)}=E+E_{\left(vs.Ag/AgCl\right)}+0.059\times pH$$

The electrolyte utilized for testing electrochemical hydrogen evolution reaction performance was a 40 mL 1 M KOH solution.

### 3.5. Benzyl Alcohol Electrocatalytic Oxidation Performance Test

This study examined the electrocatalytic oxidation of benzyl alcohol utilizing WO-N/NF as the electrocatalyst. Benzyl alcohol and its catalytic products were quantified through an Agilent 1260 high-performance liquid chromatography (HPLC) (Santa Clara, CA, USA) equipped with a UV detector. The corresponding conversion of benzyl alcohol and its product selectivity were subsequently calculated. The specific detection parameters are as follows: the liquid chromatography column was C18; the mobile phases were methanol and 10% aqueous acetic acid (the volume ratio was 4:6) at a flow rate of 0.6 mL/min; the temperature of the detection column was 40 °C; and the detection wavelength was 245 nm. The concentration of the components was quantified using the external standard method, with the peak area integral as the vertical coordinate and the concentration as the horizontal coordinate. Subsequently, the standard curve was fitted and graphed as depicted in Figure S13. The results showed that the retention times of benzyl alcohol, benzaldehyde, and benzoic acid in the mixed control solution were approximately 10 min, 14 min, and 15 min, respectively. In addition, the benzyl alcohol conversion, product selectivity, and yield were calculated using Equations (3)–(5):

(3) $$\begin{array}{c} Benzyl\;alcohol\;conversion\;\left(\%\right)=\frac{n\left(reacted\;benzyl\;alcohol\right)}{n\left(initial\;benzyl\;alcohol\right)}\times 100\% \end{array}$$

(4) $$\begin{array}{c} Benzoic\;acid\;selectivity\;\left(\%\right)=\frac{n\left(benzoic\;acid\;produced\right)}{n\left(reacted\;benzyl\;alcohol\right)}\times 100\% \end{array}$$

(5) $$\begin{array}{c} Benzoic\;acid\;yield\;\left(\%\right)=\frac{n\left(benzoic\;acid\;produced\right)}{n\left(initial\;benzyl\;alcohol\right)}\times 100\% \end{array}$$

The formula for Faradaic efficiency (FE) is given by Equation (6):

(6) $$FE=\frac{nzF}{Q}$$

where n is the actual number of moles of benzoic acid produced, z is the number of electrons transferred (4 in this reaction), F is Faraday’s constant (96,485 C/mol), and Q is the total charge passed through the circuit.

## 4. Conclusions

In summary, the N-doped bimetallic oxide electrocatalyst on Ni foam (WO-N/NF) was successfully fabricated via a one-step hydrothermal method. Owing to its optimized composition, structure, and morphology, it shows a high HER activity with potential of 1.38 V (vs. RHE) to reach a current density of 100 mA cm^−2^. The doping of tungsten induces electron transfer in WO-N/NF samples, enhancing NiOOH formation and catalytic activity for the hydrogen evolution reaction. The incorporation of nitrogen atoms into ammonium metatungstate effectively modulates the electronic structure of transition metal atoms, enhancing the catalytic performance. Furthermore, the WO-N/NF was applied to the preparation of benzoic acid by electrocatalytic oxidation of benzyl alcohol and obtained high conversion, yield, and Faradaic efficiency, reaching 98.41%, 99.66%, and 98.77%, respectively. This approach opens up new possibilities for electrocatalytic organic reactions, emphasizing the potential of our electrocatalyst in advancing green and sustainable technologies in the field.

## Figures and Tables

**Table 1:** Equivalent circuit model element parameters of NF and WO-N/NF.

Electrode	R_s_/(Ω)	R_ct_/(Ω)
NF	0.30	590.02
WO-N/NF	0.32	1.36
WO/NF	0.32	6.84
N/NF	0.33	88.52
Hydrothermal NF	0.33	97.21

## Data Availability

The data presented in this study are available in [Nitrogen-Tungsten Oxide Nanostructures on Nickel Foam as High Efficient Electrocatalysts for Benzyl Alcohol Oxidation].

## Supplementary Materials

The following supporting information can be downloaded at: https://www.mdpi.com/article/10.3390/molecules29163734/s1, Figure S1: Optical photograph of bare NF and WO-N/NF; Figure S2: SEM images of (a,b) WO/NF, (c,d) N/NF, (e,f) Hydrothermal NF; Figure S3: EDS spectrum of WO-N/NF electrode; Figure S4: XPS survey spectrum of WO-N/NF electrode; Figure S5: XPS spectrums of Ni 2p, O 1s for the obtained hydrothermal NF electrode; Figure S6: LSV curves of WO-N/NF under OER and BAO; Figure S7: Chronoamperometry tests of electrocatalytic benzyl alcohol oxidation for five cycles; Figure S8: SEM image of WO-N/NF electrode after 5 cycles of measurement; Figure S9: XRD pattern of WO-N/NF electrode after 5 cycles of measurement; Figure S10: XPS spectrums of Ni 2p (a), W 4f (b), O 1s (c), and N 1s (d) for the obtained WO-N/NF electrode after 5 cycles of measurement; Figure S11: First-order kinetics models of the electrochemical oxidation of BAO for WO-N/NF and NF at 1.87 V vs. RHE; Figure S12: Scheme for the synthesis of WO-N/NF electrocatalyst for benzyl alcohol oxidation; Figure S13: HPLC chromatogram of the standard mixed solution at different concentrations (a) benzyl alcohol, (c) benzaldehyde and (e) benzoic acid. The standard curves of (b) benzyl alcohol, (d) benzaldehyde and (f) benzoic acid; Table S1: Composition and content of each element in WO-N/NF electrode; Table S2: Comparison of the catalytic performance of the WO-N/NF and the stand-of-the-art catalysts towards benzyl alcohol electrochemical oxidation; Table S3: Each abbreviation corresponds to the full name; Synthesis of WO/NF; Synthesis of N/NF; Synthesis of hydrothermal NF. References [15,17,58,59,60,61,62,63,64,65,66,67] are cited in Supplementary Materials file.
