# Supplementary material for: Nitrogen-Tungsten Oxide Nanostructures on Nickel Foam as High Efficient Electrocatalysts for Benzyl Alcohol Oxidation
Source: Molecules. 2024 Aug 7;29(16):3734. doi: 10.3390/molecules29163734 (PMC11357156; doi:10.3390/molecules29163734)
Supplement: Supplementary file 1 [file molecules-29-03734-s001.zip › molecules-3053510-supplementary.pdf]

Supporting Information

Nitrogen-tungsten oxide nanostructures on nickel foam as high efficient electrocatalysts for benzyl alcohol oxidation

Yizhen Zhu,[a,b] Xiangyu Chen,[a,b] Yuanyao Zhang,[a] Zhifei Zhu,[a] Handan Chen,[a] Kejie Chai,[a] * and Weiming Xu [a] *

[a] *College of Material, Chemistry and Chemical Engineering, Hangzhou Normal University, Hangzhou 311121, China.*

[b] *Kharkiv Institute, Hangzhou Normal University, Hangzhou 311121, China.*

Corresponding Authors:

Prof. Weiming Xu

Email: wmxu@zju.edu.cn

Dr. Kejie Chai

Email: kjchai@foxmail.com

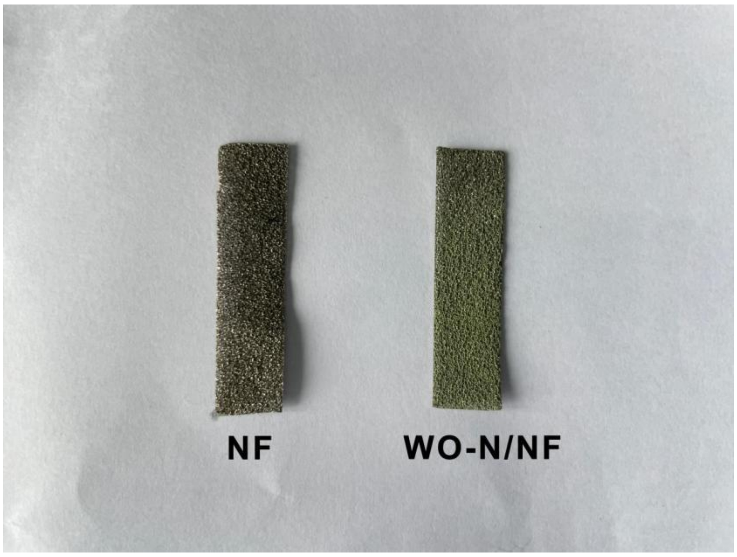

Figure S1. Optical photograph of bare NF and WO-N/NF.

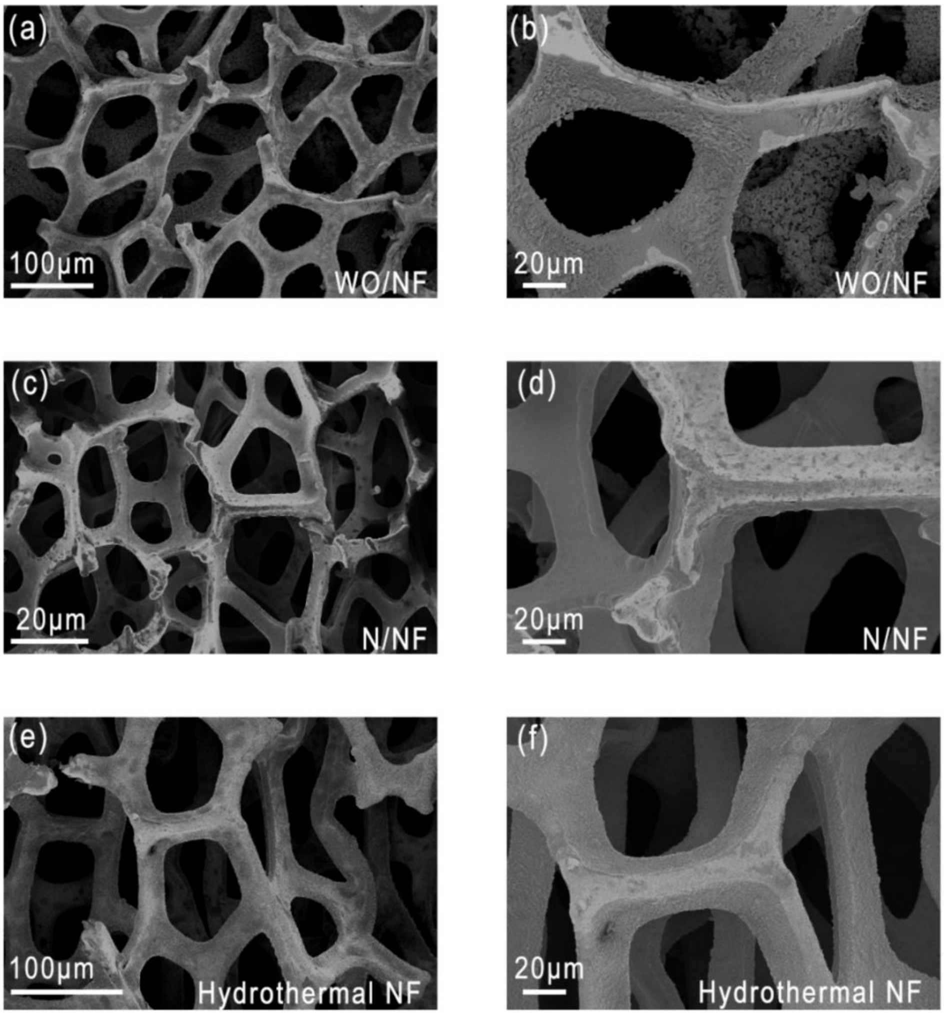

Figure S2. SEM images of (a, b) WO/NF, (c, d) N/NF, (e, f) Hydrothermal NF.

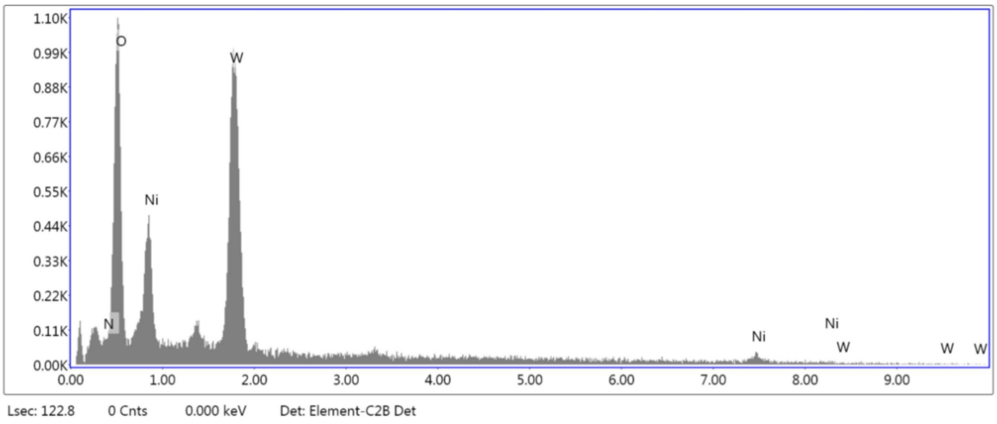

Figure S3. EDS spectrum of WO-N/NF electrode.

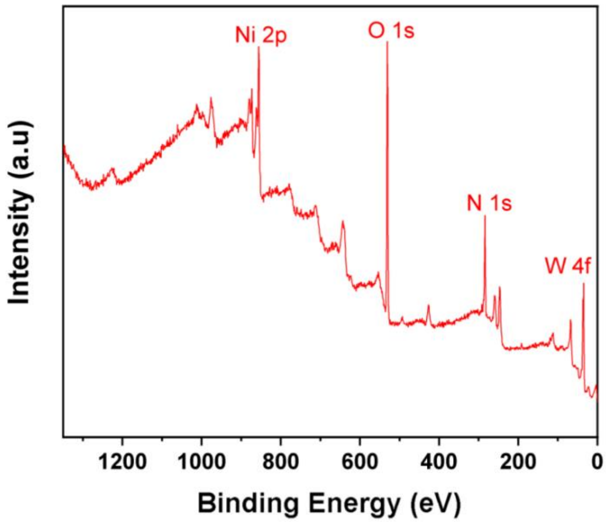

Figure S4. XPS survey spectrum of WO-N/NF electrode.

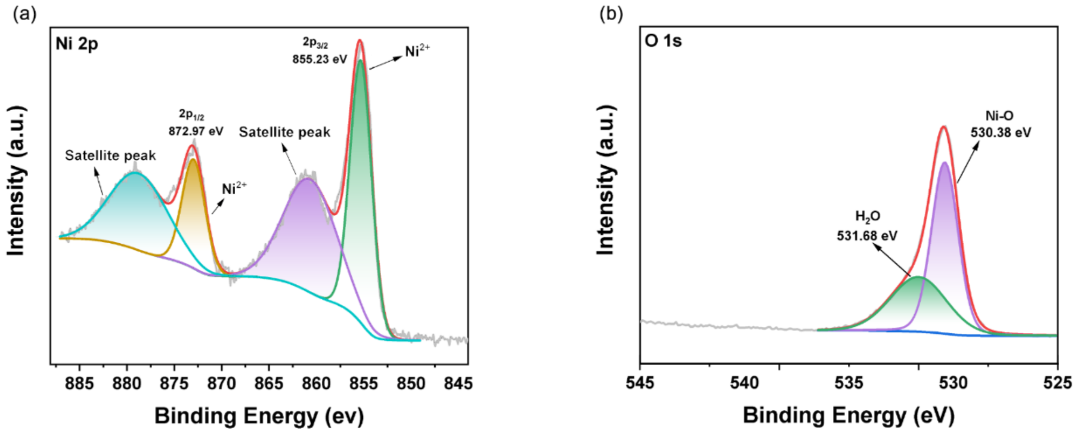

Figure S5. XPS spectrums of Ni 2p, O 1s for the obtained hydrothermal NF electrode.

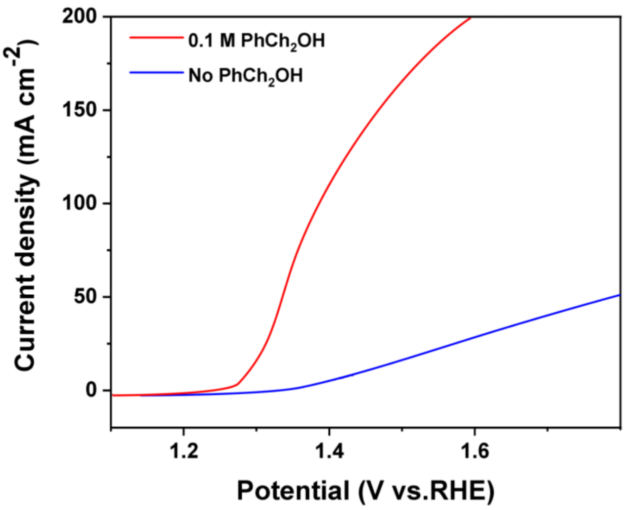

Figure S6. LSV curves of WO-N/NF under OER and BAO.

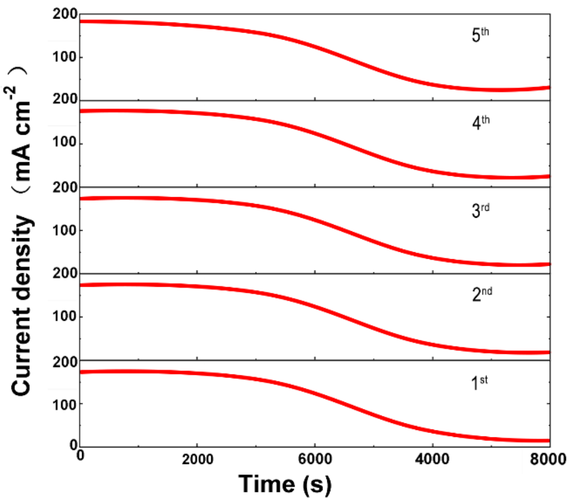

Figure S7. Chronoamperometry tests of electrocatalytic benzyl alcohol oxidation for five cycles.

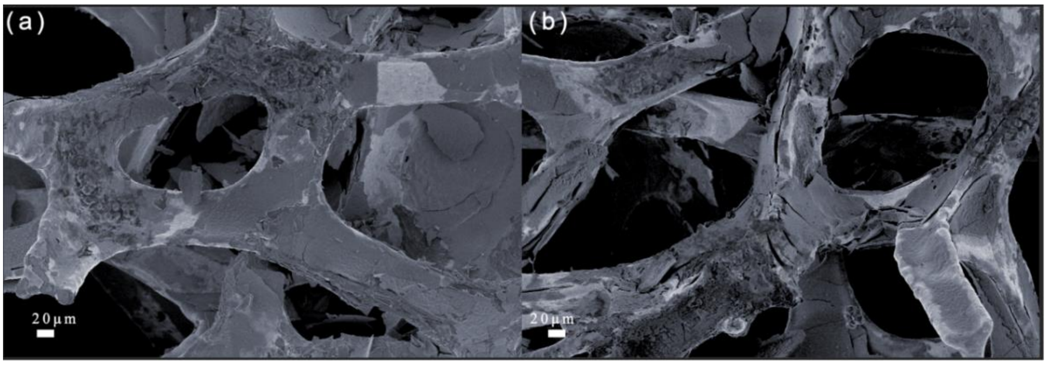

Figure S8. SEM image of WO-N/NF electrode after 5 cycles of measurement.

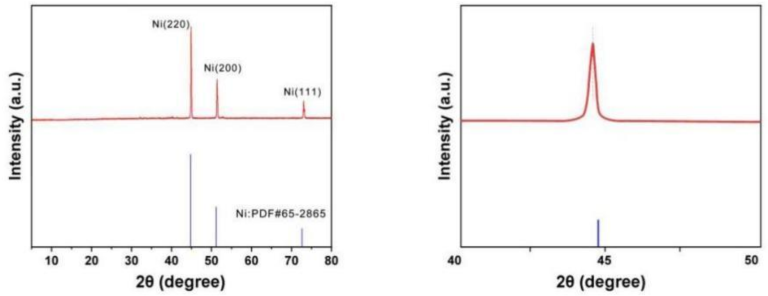

Figure S9. XRD pattern of WO-N/NF electrode after 5 cycles of measurement.

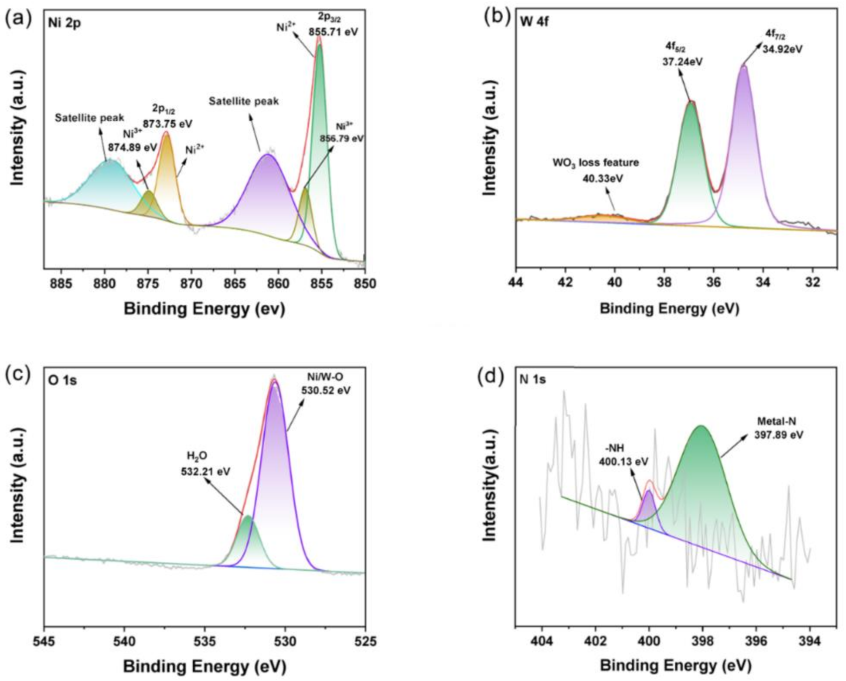

Figure S10. XPS spectrums of Ni 2p (a), W 4f (b), O 1s (c), and N 1s (d) for the obtained WO-N/NF electrode after 5 cycles of measurement.

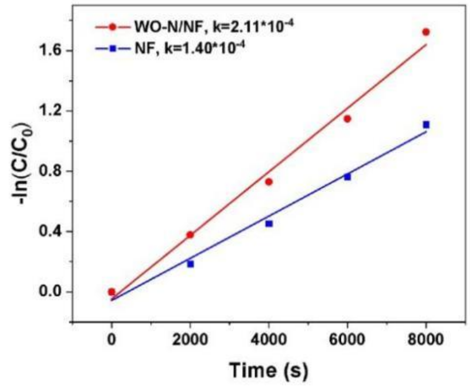

Figure S11. First-order kinetics models of the electrochemical oxidation of BAO for WO-N/NF and NF at 1.87 V vs. RHE.

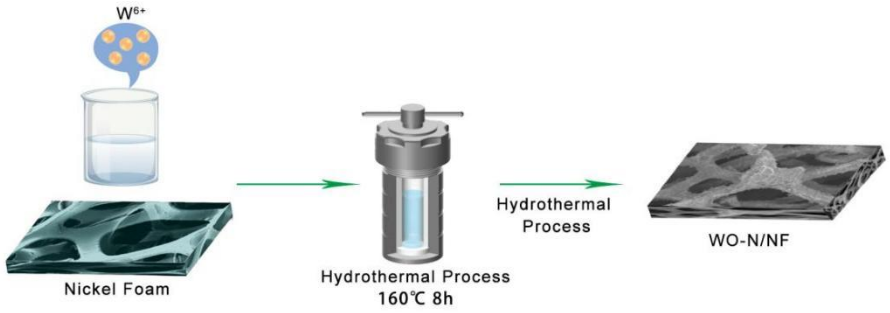

Figure S12. Scheme for the synthesis of WO-N/NF electrocatalyst for benzyl alcohol oxidation.

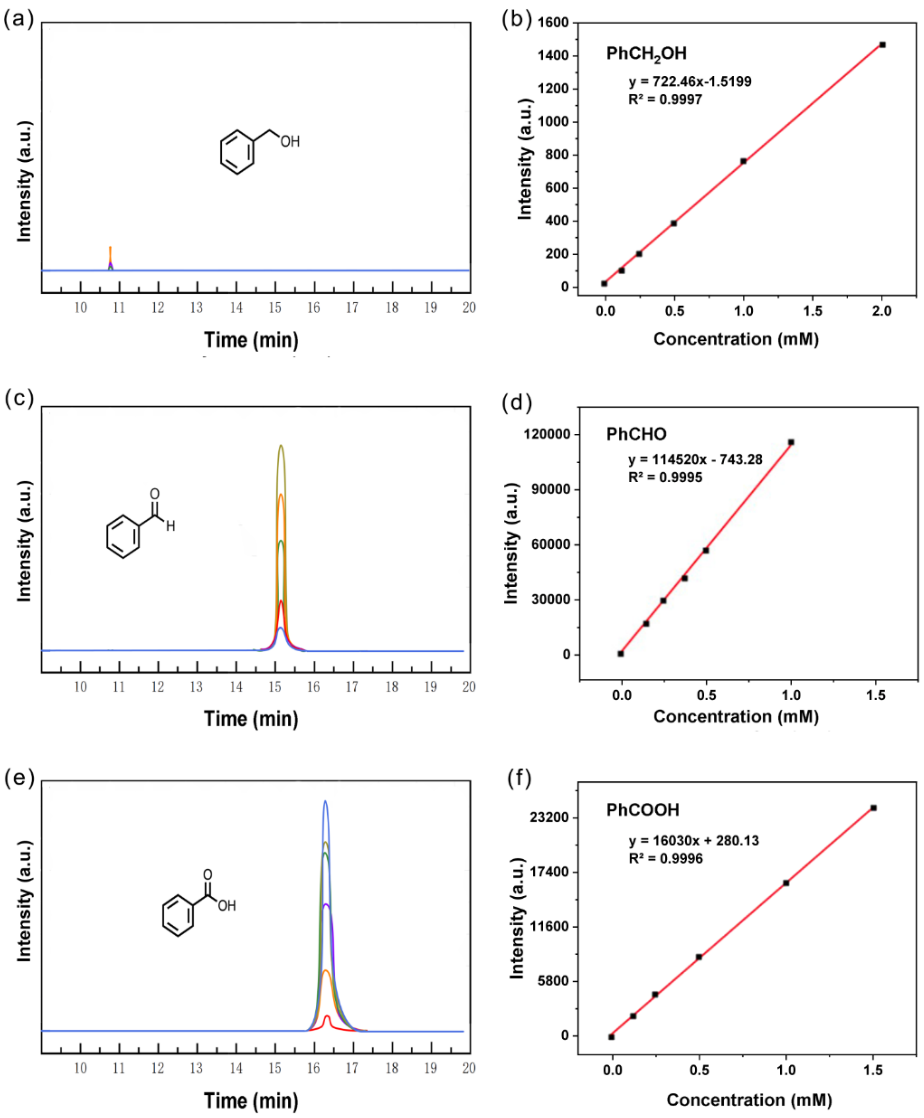

Figure S13. HPLC chromatogram of the standard mixed solution at different concentrations (a) benzyl alcohol, (c) benzaldehyde and (e) benzoic acid. The standard curves of (b) benzyl alcohol, (d) benzaldehyde and (f) benzoic acid.

Table S1. Composition and content of each element in WO-N/NF electrode.

Element	Weight %	Atomic %	Error %
N	0.43	1.40	73.56
O	25.61	72.33	8.19
Ni	15.45	11.89	6.58
W	58.50	14.38	4.98

Table S2. Comparison of the catalytic performance of the WO-N/NF and the stand-of-the-art catalysts towards benzyl alcohol electrochemical oxidation.

Electrocatalyst	Electrolyte	Benzyl alcohol concentration (mM)	Potential @ Current density	Convesion (%)	Faradic Efficiency (%)	Ref.
WO-N/NF	1M KOH	100	1.38@100	99.7	98.8	This work
$NiCo_2O_4$/NF	1M KOH	50	1.46@100	95	99	1
NC@$CuCo_2N_x$/CF	1M KOH	15	1.25@10	97	81.4	2
Co_3O_4/NF	1M KOH	20	1.50@86	>99	>99	3
Co300	1M KOH	20	1.50@37	71.7	67.8	3
A-Ni-Co-H/NF	1M KOH	100	1.35@100	99.6	93.5	4
Co_3O_4 NWs/Ti	0.1M NaOH	50	2.1@2.6	99.2	/	5
NC@CuCo	1M KOH	15	1.25@10	97.5	81.3	6
Ni-OH/NF	1M NaOH	100	~1.33@100	84.5	99	7
h-$Ni(OH)_2$	1M KOH	40	1.45@100	99.5	98.6	8
Mo-Ni	1M KOH	10	1.53@100	99	96.5	9
hp-Ni	1M KOH	10	1.40@100	/	98	10
Fe/Co200	1M KOH	15	1.438@10	/	99.4	11
NiCo-21-MOF	1M KOH	100	1.52@295.48	86	/	12

Table S3. Each abbreviation corresponds to the full name.

Abbreviation	Full name
WO-N/NF	Nitrogen-doped Tungsten-nickel Bimetallic Oxide Electrocatalyst
OER	Oxygen Evolution Reaction
HER	Hydrogen Evolution Reaction
TMTs	Transition Metal Tungstates
HPLC	High Performance Liquid Chromatography
XRD	X-Ray Diffraction
SEM	Scanning Electron Microscope

TEM	Transmission Electron Microscope
HRTEM	High Resolution Transmission Electron Microscope
SAED	Selected Area Electron Diffraction
XPS	X-Ray Photoelectron Spectroscopy
NF	Nickel Foam
WO-/NF	Tungsten-nickel Bimetallic Oxide Electrocatalyst
N/NF	Nitrogen-doped Nickel Metallic Oxide Electrocatalyst
ECSA	Electrochemical Active Surface Area
RHE	Reversible Hydrogen Electrode

Synthesis of WO/NF

The materials were prepared using a one-step hydrothermal method. A 10×40×1 mm^3 nickel foam was initially intercepted and then ultrasonically washed in sequence using acetone, 1 M hydrochloric acid, and deionized water for 30 min. Subsequently, the foam was vacuum-dried. 0.2 mmol $NaWO_3$ and 0.1 mmol $NiC_4H_6O_4\cdot4H_2O$ were dissolved in 40 mL deionized water by stirring at room temperature using a stirring table. The configured solution and nickel foam were moved to a 100 mL hydrothermal reactor and heated in an oven at 160 °C for 8 h. Then cooled to room temperature, the nickel foam with color change was collected, rinsed with deionized water to remove the impurities on the surface, and then dried at 50 °C in a vacuum oven for 12 h to obtain the WO/NF.

Synthesis of N/NF

The materials were prepared using a one-step hydrothermal method. A 10×40×1 mm^3 nickel foam was initially intercepted and then ultrasonically washed in sequence using acetone, 1 M hydrochloric acid, and deionized water for 30 min. Subsequently, the foam was vacuum-dried. 0.2 mmol melamine was dissolved in 40 mL deionized water by stirring at room temperature using a stirring table. The configured solution and nickel foam were moved to a 100 mL hydrothermal reactor and heated in an oven at 160 °C for 8 h. Then cooled to room temperature, the nickel foam with color change was

collected, rinsed with deionized water to remove the impurities on the surface, and then dried at 50 °C in a vacuum oven for 12 h to obtain the N/NF.

Synthesis of hydrothermal NF

The materials were prepared using a one-step hydrothermal method. A 10×40×1 mm^3 nickel foam was initially intercepted and then ultrasonically washed in sequence using acetone, 1 M hydrochloric acid, and deionized water for 30 min. Subsequently, the foam was vacuum-dried. 40 mL deionized water and nickel foam were moved to a 100 mL hydrothermal reactor and heated in an oven at 160 °C for 8 h. Then cooled to room temperature, the nickel foam with color change was collected, rinsed with deionized water to remove the impurities on the surface, and then dried at 50 °C in a vacuum oven for 12 h to obtain the hydrothermal NF.

Reference

[1] M. Xu, J. Geng, H. Xu, S. Zhang, H. Zhang, In situ construction of $NiCo_2O_4$ nanosheets on nickel foam for efficient electrocatalytic oxidation of benzyl alcohol, Inorganic Chemistry Frontiers, 10 (2023) 2053-2059.

[2] J. Zheng, X. Chen, X. Zhong, S. Li, T. Liu, G. Zhuang, X. Li, S. Deng, D. Mei, J.-G. Wang, Hierarchical Porous NC@CuCo Nitride Nanosheet Networks: Highly Efficient Bifunctional Electrocatalyst for Overall Water Splitting and Selective Electrooxidation of Benzyl Alcohol, Advanced Functional Materials, 27 (2017) 1704169.

[3] Y. Cao, D. Zhang, X. Kong, F. Zhang, X. Lei, Multi-vacancy Co_3O_4 on nickel foam synthesized via a one-step hydrothermal method for high-efficiency electrocatalytic benzyl alcohol oxidation, Journal of Materials Science, 56 (2021) 6689-6703.

[4] H. Huang, C. Yu, X. Han, H. Huang, Q. Wei, W. Guo, Z. Wang, J. Qiu, Ni, Co hydroxide triggers electrocatalytic production of high-purity benzoic acid over 400 mA cm^{-2}, Energy & Environmental Science, 13 (2020) 4990-4999.

[5] Z. Yin, Y. Zheng, H. Wang, J. Li, Q. Zhu, Y. Wang, N. Ma, G. Hu, B. He, A. Knop-Gericke, R. Schlögl, D. Ma, Engineering Interface with One-Dimensional Co_3O_4 Nanostructure in Catalytic Membrane Electrode: Toward an Advanced Electrocatalyst for Alcohol Oxidation, ACS Nano, 11 (2017) 12365-12377.

[6] L. Ming, X.-Y. Wu, S.-S. Wang, W. Wu, C.-Z. Lu, Facile growth of transition metal hydroxide nanosheets on porous nickel foam for efficient electrooxidation of benzyl alcohol, Green Chemistry, 23 (2021) 7825-7830.

[7] X. Chen, X. Zhong, B. Yuan, S. Li, Y. Gu, Q. Zhang, G. Zhuang, X. Li, S. Deng, J.-g. Wang, Defect engineering of nickel hydroxide nanosheets by Ostwald ripening for enhanced selective electrocatalytic alcohol oxidation, Green Chemistry, 21 (2019) 578-588.
[8] B. You, X. Liu, X. Liu, Y. Sun, Efficient H_2 Evolution Coupled with Oxidative Refining of Alcohols via A Hierarchically Porous Nickel Bifunctional Electrocatalyst, ACS Catalysis, 7 (2017) 4564-4570.
[9] X. Cui, M. Chen, R. Xiong, J. Sun, X. Liu, B. Geng, Ultrastable and efficient H_2 production via membrane-free hybrid water electrolysis over a bifunctional catalyst of hierarchical Mo–Ni alloy nanoparticles, Journal of Materials Chemistry A, 7 (2019) 16501-16507.
[10] Y. Huang, R. Yang, G. Anandhababu, J. Xie, J. Lv, X. Zhao, X. Wang, M. Wu, Q. Li, Y. Wang, Cobalt/Iron(Oxides) Heterostructures for Efficient Oxygen Evolution and Benzyl Alcohol Oxidation Reactions, ACS Energy Letters, 3 (2018) 1854-1860.
[11] Y. Song, M. Yuan, W. Su, D. Guo, X. Chen, G. Sun, W. Zhang, Ultrathin Two-Dimensional Bimetal–Organic Framework Nanosheets as High-Performance Electrocatalysts for Benzyl Alcohol Oxidation, Inorganic Chemistry, 61 (2022) 7308-7317.
[12] C. Zhang, S. Ci, X. Peng, J. Huang, P. Cai, Y. Ding, Z. Wen, Tri-profit electrolysis for energy-efficient production of benzoic acid and H_2, Journal of Energy Chemistry, 54 (2021) 30-35.
